# A Low-Pressure, N_2_/CO_2_ Atmosphere Is Suitable for Cyanobacterium-Based Life-Support Systems on Mars

**DOI:** 10.3389/fmicb.2021.611798

**Published:** 2021-02-16

**Authors:** Cyprien Verseux, Christiane Heinicke, Tiago P. Ramalho, Jonathan Determann, Malte Duckhorn, Michael Smagin, Marc Avila

**Affiliations:** Center of Applied Space Technology and Microgravity (ZARM), University of Bremen, Bremen, Germany

**Keywords:** life-support systems, space exploration, ISRU, CyBLiSS, hypobaria, low-pressure microbiology

## Abstract

The leading space agencies aim for crewed missions to Mars in the coming decades. Among the associated challenges is the need to provide astronauts with life-support consumables and, for a Mars exploration program to be sustainable, most of those consumables should be generated on site. Research is being done to achieve this using cyanobacteria: fed from Mars's regolith and atmosphere, they would serve as a basis for biological life-support systems that rely on local materials. Efficiency will largely depend on cyanobacteria's behavior under artificial atmospheres: a compromise is needed between conditions that would be desirable from a purely engineering and logistical standpoint (by being close to conditions found on the Martian surface) and conditions that optimize cyanobacterial productivity. To help identify this compromise, we developed a low-pressure photobioreactor, dubbed Atmos, that can provide tightly regulated atmospheric conditions to nine cultivation chambers. We used it to study the effects of a 96% N_2_, 4% CO_2_ gas mixture at a total pressure of 100 hPa on *Anabaena* sp. PCC 7938. We showed that those atmospheric conditions (referred to as MDA-1) can support the vigorous autotrophic, diazotrophic growth of cyanobacteria. We found that MDA-1 did not prevent *Anabaena* sp. from using an analog of Martian regolith (MGS-1) as a nutrient source. Finally, we demonstrated that cyanobacterial biomass grown under MDA-1 could be used for feeding secondary consumers (here, the heterotrophic bacterium *E. coli* W). Taken as a whole, our results suggest that a mixture of gases extracted from the Martian atmosphere, brought to approximately one tenth of Earth's pressure at sea level, would be suitable for photobioreactor modules of cyanobacterium-based life-support systems. This finding could greatly enhance the viability of such systems on Mars.

## 1. Introduction

The Global Exploration Roadmap issued by the International Space Exploration Coordination Group, a forum gathering over 20 space agencies, lists crewed missions to Mars as a common driving goal (ISECG, 2018). It is reflected in the plans of individual agencies: as a notable example, NASA, supported by others such as CSA, ESA, Roscosmos, and JAXA, aims at returning to the Moon by 2024 and establishing a sustainable presence there by 2028 (NASA, 2019). This endeavor is a milestone in a larger program leading to crewed missions to Mars, tentatively planned for the 2030s (115^*th*^ Congress of the USA, 2017; Trump, 2017; NASA, 2020). Private companies have stated related goals; chiefly, SpaceX aims for Mars landings as early as the 2020s (Musk, 2017). While timelines are likely to be revised, crewed missions to Mars may take place in the coming decades.

Among the associated challenges is the need to provide crews with life-support consumables. Those for the first mission may be sent off Earth, but launch costs, travel times, and risks of failure are such that the viability of a sustainable program will depend on our ability to produce consumables on site (Horneck et al., 2006). Life sciences may support this ability: biological systems for the production and recycling of essential resources, referred to as bioregenerative life-support systems (BLSS), have been proposed for spaceflight and planetary outposts (see for instance Gòdia et al., 2002; Lobascio et al., 2007; Nelson et al., 2010). Some include cyanobacteria. *Limnospira indica*, for instance, is being considered for air revitalization, nitrate removal and edible biomass production in the Micro-Ecological Life Support System Alternative (MELiSSA), a BLSS project aimed at regenerating atmospheric gases, recycling water, treating waste, and producing food for crewed space missions (Gòdia et al., 2002; Poughon et al., 2020). The use of desert isolates has been suggested as well, based on the assumption that extremophilic features may be an advantage in case of exposure to harsh environmental conditions (Billi et al., 2017; Billi, 2019).

**Figure 1:**
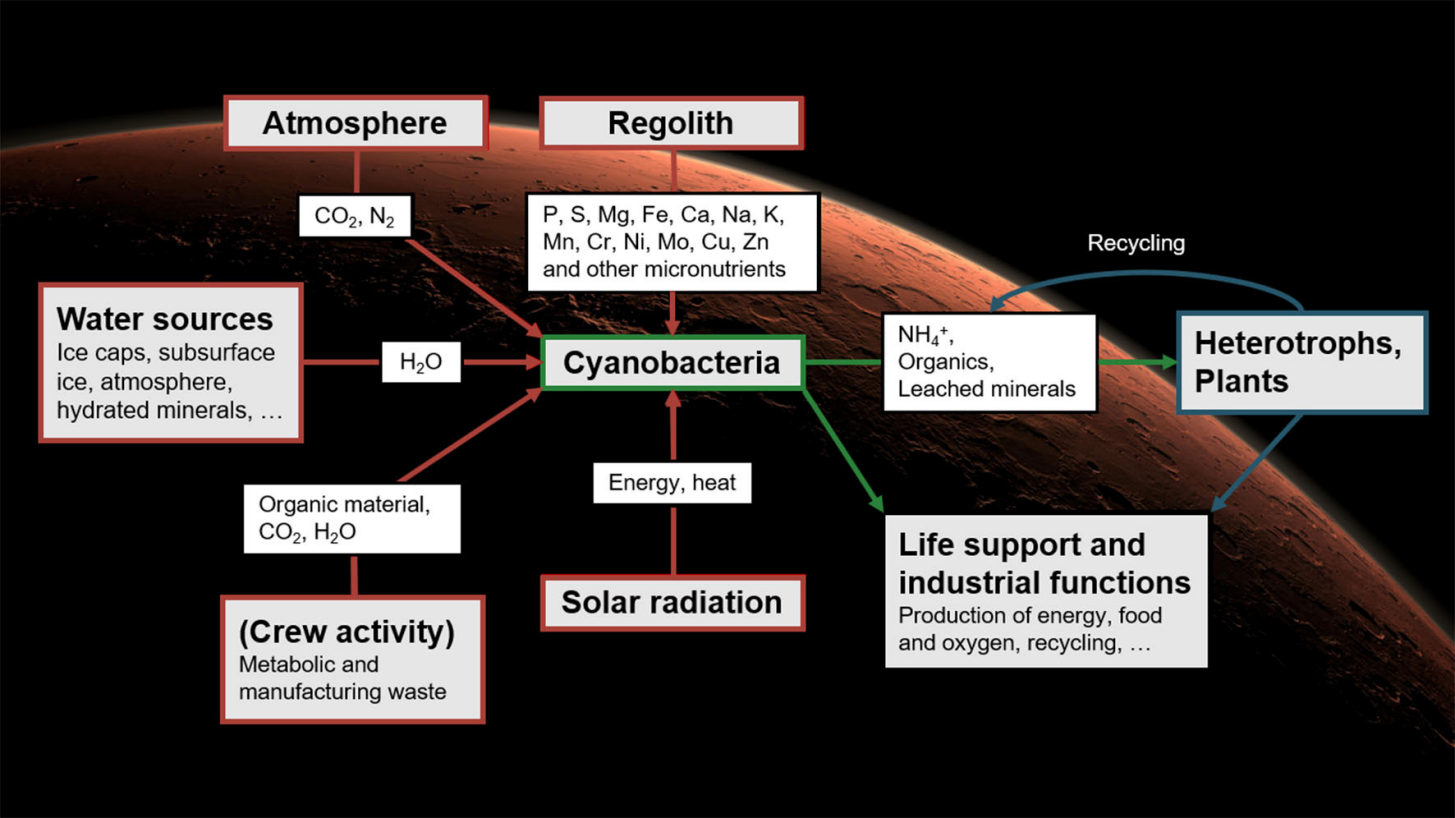
Cyanobacterium-based life-support systems on Mars: using cyanobacteria fed with local resources to decrease the dependence on Earth-imported materials of future long-duration, crewed missions. The concept was described in detail by Verseux et al. (2016a). Reproduced from Verseux (2020b) with permission from the editor (Figure 12.1, p. 290).

Relying exclusively on materials imported from Earth would limit the autonomy of BLSS: without resupply, the amounts of elements in the system could only decrease over time. While not a major obstacle in low Earth orbit, this would be unsuitable for long-term stays on Mars. There, diazotrophic, rock-weathering cyanobacteria may play a central role: it has been argued that they could be used as a basis for BLSS that would rely on local resources, thereby greatly reducing the crew's dependence on Earth (Brown et al., 2008; Olsson-Francis and Cockell, 2010; Verseux et al., 2016a). First, the cyanobacteria would be fed with materials available on site: water could be mined from the ground and atmosphere; carbon and nitrogen (available as CO_2_ and N_2_) could feed their photosynthetic and diazotrophic metabolism; and all other required nutrients are present in the regolith (Cockell, 2014) and could, it seems, be exploited by species endowed with abilities to process basaltic substrates (Brown et al., 2010; Olsson-Francis and Cockell, 2010; Verseux et al., 2016a). The cultured cyanobacteria could produce various consumables directly (such as O_2_ and dietary proteins) but also support the growth of other organisms (Verseux et al., 2016a; Verseux, 2018). The secondary producers could then synthesize further resources (Hendrickx and Mergeay, 2007; Brown et al., 2008; Rothschild, 2016; Verseux et al., 2016a,b), and genetic engineering could increase both efficiency and the range of applications (Verseux et al., 2016b). An overview of a concept for cyanobacterium-based life-support systems (CyBLiSS) is given in Figure 1.

Among the factors that will determine the efficiency of CyBLiSS is the physiology of cyanobacteria under non-Earth atmospheres (Verseux et al., 2016a; Verseux, 2020a). In principle, cultivating cyanobacteria under atmospheric conditions close to Mars's would offer several advantages. The low pressure would decrease constraints on robustness: a wider range of materials could be used for the photobioreactor (including, for instance, materials transparent to photosynthetically active radiation) and the mass of structural materials could be reduced. It would also help lower the rates of leakage, reducing both the amounts of consumables to be replenished and the risk of outward biological contamination (Boston, 1981; Lehto et al., 2006; Richards et al., 2006). Relying on a gas composition close to Mars's would, in addition, facilitate the utilization of the local atmosphere.

However, cyanobacteria could not thrive under Mars-ambient atmospheric conditions. First, the total pressure is too low: on-site surface measurements have varied between approximately 6 and 11 hPa (sol average), with large seasonal and diurnal variations (Harri et al., 2014; Martínez et al., 2017). Such values are incompatible with the metabolism of most microorganisms (Schwendner and Schuerger, 2020; Verseux, 2020a), as well as with the stability of liquid water at temperatures which are supportive of cyanobacterial growth. Second, the fraction represented by N_2_ is too low for diazotrophic growth at a low total pressure; results from the Sample Analysis at Mars (SAM) instrument suite on Curiosity indicate that Mars's atmosphere contains circa 95% CO_2_, 2.8% N_2_, 2.1% Ar, and trace gases (Franz et al., 2017), which is close to values obtained from the Viking mission (Owen et al., 1977; Oyama and Berdahl, 1977). This raises the following question: How close can a photobioreactor's gas phase be to Mars's atmosphere while enabling the vigorous auto- and diazotrophic growth of selected cyanobacteria?

Although the effects on microorganisms of variations in atmospheric conditions remain poorly understood, a total pressure down to about 100 hPa (rather than ambient, sea-level pressure) is not expected to largely affect, *per se*, microbial growth (Schuerger et al., 2013; Verseux, 2020a). Lower values could be considered but the partial pressures of gaseous carbon and nitrogen, which must each be high enough to sustain metabolism, set a lower limit. Evidence suggests that for at least some species of cyanobacteria, the partial pressure of CO_2_ (pCO_2_) is non-limiting from ca. 4 hPa on, and those species can grow under low pressures of close-to-pure CO_2_ if all other required nutrients (notably, a source of nitrogen) are provided in the culture medium (Murukesan et al., 2015). N_2_, on the other hand, was shown to be limiting down from a partial pressure of around 500 hPa for various nitrogen-fixing bacteria (MacRae, 1977; Klingler et al., 1989; Silverman et al., 2019), though growth of *Anabaena cylindrica* and *A. variabilis* was still vigorous at a pN_2_ of 100 hPa under ambient pressure (Silverman et al., 2019). Thus, the lowest pressure that can be used in a CyBLiSS photobioreactor on the Martian surface seems most constrained by pN_2_.

**Figure 2:**
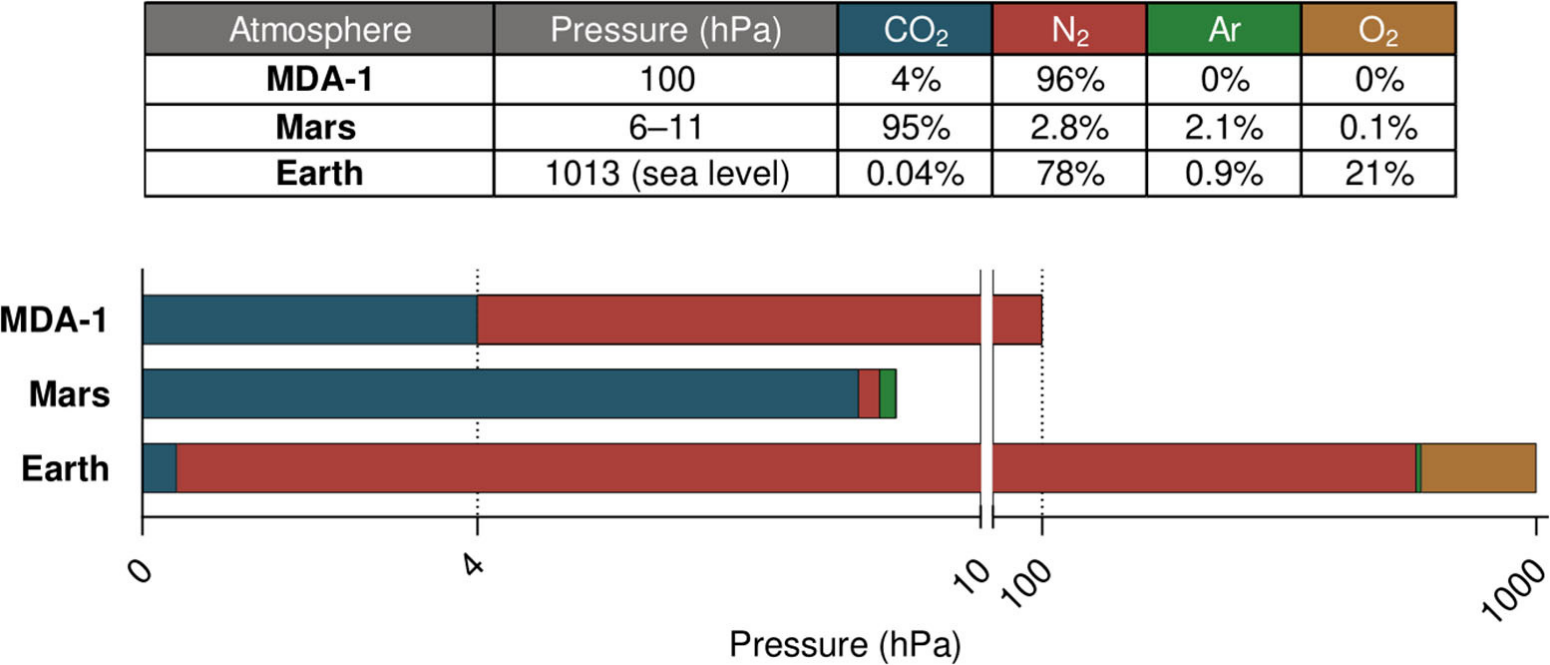
Comparison of Earth, Mars, and MDA-1 atmospheres. The scale of the bar graph is linear over each of both segments (0–10 and 10–1,020 hPa). Gas proportions in the table are given in volume percent; data for Mars are from Franz et al. (2017), those for Earth from NOAA et al. (1976) and NOAA (2020).

In the work reported here, we used a low-pressure, atmosphere-controlled photobioreactor developed in-house to study the impact on cyanobacterial cultures of a 96% N_2_, 4% CO_2_ mixture at 100 hPa (see Figure 2 for a comparison with Earth's and Mars's atmospheres). Due to its low pressure and its composition derived from gases available on Mars, this atmosphere (hereafter referred to as MDA-1, for Mars-derived atmosphere 1) would greatly reduce the engineering and logistical constraints of a Martian photobioreactor. Based on the considerations given above on the biological effects of total pressure, pCO_2_ and pN_2_, we hypothesized that MDA-1 would be suitable for diazotrophic cyanobacterial growth: the pCO_2_ is presumably non-limiting and more supportive of growth than Earth-ambient pCO_2_; the total pressure of 100 hPa is thought not to largely affect bacteria; and a compromise was made for the pN_2_, non-limiting levels of which would conflict with the requirement of maintaining a low total pressure.

We first tested whether MDA-1 could support the vigorous, diazotrophic growth of *Anabaena* sp. in a standard medium. We then assessed whether it would prevent this cyanobacterium from relying on regolith in water for all nutrients not provided as gases. Finally, we determined whether nutrients extracted from cyanobacteria grown under MDA-1 could support downstream BLSS modules. Taken as a whole, our results suggest that a low-pressure, N_2_/CO_2_ atmosphere would be suitable for use in a CyBLiSS photobioreactor. This could greatly improve the feasibility of such systems on Mars.

## 2. Materials and Methods

### 2.1. Bacterial Strains

*Anabaena* sp. PCC 7938 (hereafter *Anabaena* sp.) was obtained from the Pasteur Culture Collection of Cyanobacteria (Paris, France). It was routinely grown in BG11_0_ at 25 °C in a poly klima PK 520-LED growth chamber, under 10–15 μmol photons m^−2^ s^−1^, with a 16 h/8 h day/night cycle.

*Escherichia coli* W (DSM 1116) was obtained from the German Collection of Microorganisms and Cell Cultures (Braunschweig, Germany). Prior to experiments, samples from glycerol stocks were streaked on LB-agar and incubated overnight at 37 °C.

### 2.2. Low-Pressure Photobioreactor (Atmos)

**Figure 3:**
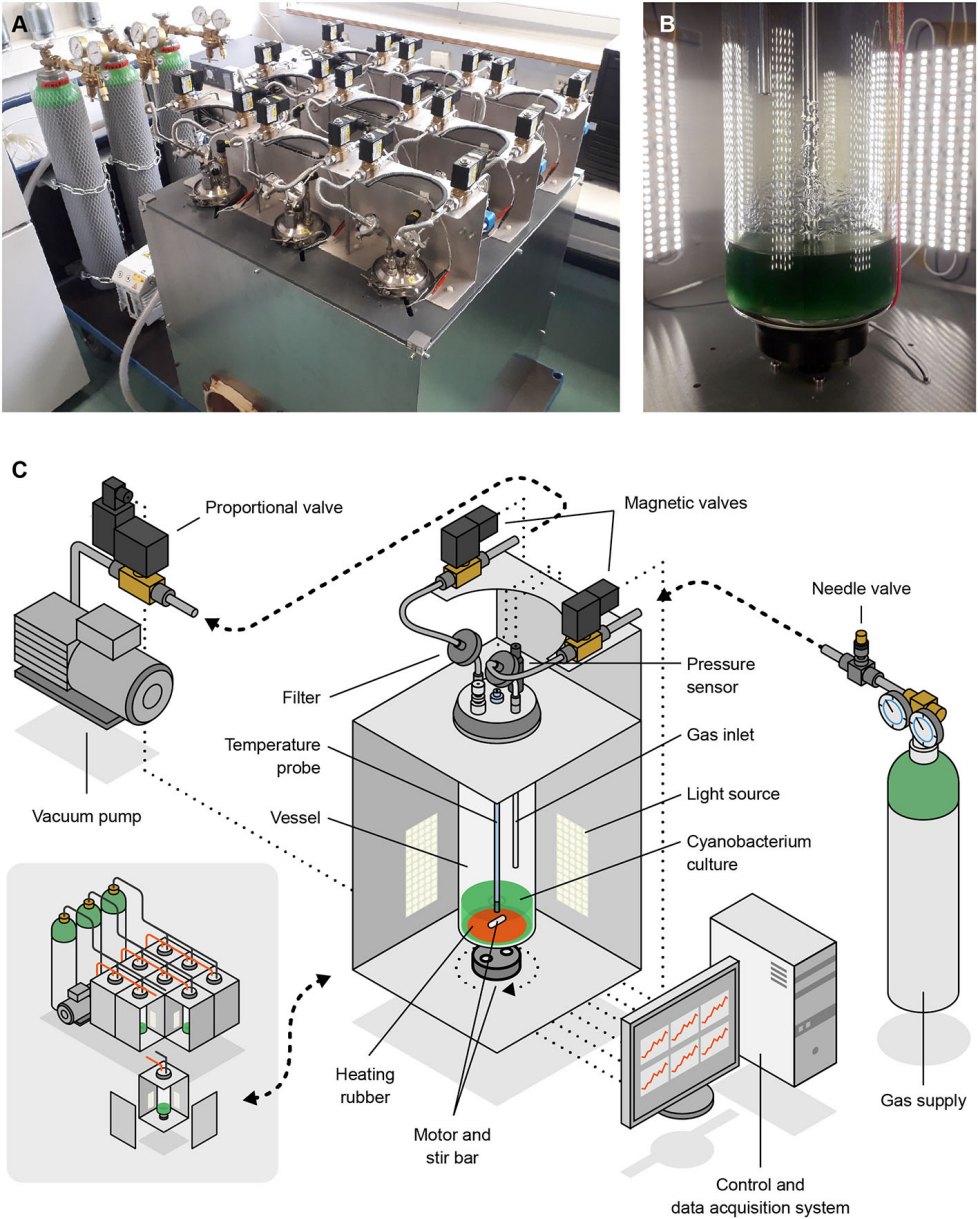
Photographs **(A,B)** and schemes **(C)** of Atmos, the low-pressure photobioreactor we developed and used in this study. Shown are an overview of the device (**A**; bottom-left section of **C**) and the inside of one of the 9 vessel compartments (**B**; main section of **C**). Artwork by Joris Wegner (University of the Arts Bremen).

Our study relied on a low-pressure, atmosphere-controlled photobioreactor which we dubbed Atmos (standing for Atmosphere Tester for Mars-bound Organic Systems). This device (see Figure 3) comprises nine vessels, each of which can host up to 1.17 l (including the gas phase) of a photosynthetic microbial culture, providing 4-sided illumination, stirring, heating and, most notably, accurately controlled atmospheric conditions. Each row of 3 vessels can be connected to a separate gas source, and each vessel filled up to a different pressure. The system is software-controlled and all actions needed throughout cultivation (e.g., adjusting and recording pressure and temperature, and renewing the gas phase at defined intervals) are automated. We developed Atmos in-house as no photobioreactor was available that featured an accurate control of atmospheric conditions at 100 hPa or below. Other functionalities are currently being developed for use in future experiments.

Each vessel is a 6.4 cm diameter glass cylinder covered with a stainless steel lid which is held in place by a clamping ring and sealed with an O-ring. The lid has five G1/4” holes, two of which host the gas inlet (black lines in Figure 3C) and outlet (red lines), and two others the temperature and pressure probes described below (the fifth hole can be sealed). Gas coming into and out of the vessels is filtered through 0.20 μm polytetrafluoroethylene membranes (Sartorius). All the inner parts of the vessels, in-between the filters, can be autoclaved, except for the temperature and pressure probes. Those are disinfected by incubation in 70% ethanol before being mounted into the lid (under a laminar flow hood).

All vessels are illuminated with OPTONICA ST4763 LED-strips, distributed in four groups per vessel (one on each side) of 5 13-cm, 27-LED strips. Each set of 5 strips provides an illumination of up to 825 lm per m of strip, at a color temperature of 2,800 K. The photon flux density was calibrated using Apogee Instruments' MQ-200 quantum sensor, holding the hand-held meter against the inner wall of a vessel.

At the bottom of each vessel, a 51 mm diameter Minco HR6939 silicone rubber heater is glued to the glass that can transfer 20 W at 24 V. The temperature of the medium is monitored with a PT100 (accuracy class DIN1/3) that is screwed into one of the G1/4 holes. The probe has a length of 375 mm and reaches down to approximately 1.5 cm above the bottom of the vessel.

Cultures can be stirred with magnetic stirrers; we use a stir bar inside each vessel and an ACT 11HS5406 stepper motor underneath that needs 200 steps for completing one full rotation. All motors are controlled by the Emis SMC-1000i stepper motor controller via a USB connection. A small 3D-printed adapter is attached to the motor shaft to hold 2 cylindrical, neodymium magnets of 10 mm diameter.

For pressure regulation, we use a Leybold D4B, a two-stage oil-sealed rotary vane vacuum pump. It has a constant pumping speed of 4.2 m^3^ h^−1^ and could attain a final pressure of 3 × 10^−3^ hPa. Pressure inside the vessels is measured with a Baumer PBMN, which has a range of 0–1.6 bar (160 kPa), a standard error of 0.04% of the full-scale range, and can be used for real-time measurements. Similar to the temperature sensor, it is screwed into one of the holes in the lid, but it does not reach the water surface.

The vacuum pump is always active; actual gas flow into and out of a vessel (when changing the pressure or renewing the gas phase) is controlled by three types of valves: needle valves, magnetic valves, and proportional valves. One needle valve per row (SS-SS6MM-VH, Swagelok) controls the inflow into the vessel row; one proportional valve per row (SCG202A053V) regulates the outflow from the vessel row; and two magnetic valves per vessel (Buschjost GP1625611) determine which vessel of the row is being regulated.

All measurement devices and actuators are connected to a computer and managed using LabVIEW. The program consists of five modules: control, user actions (e.g., changing cultivation parameters or starting an experiment), data acquisition, memory access, and graphic visualization.

### 2.3. Cultivation of *Anabaena* sp. Under MDA-1 in Standard Medium

A culture of *Anabaena* sp. in late exponential phase was used to inoculate six vessels filled with BG11_0_ (2 of the 3 remaining vessels were used for the regolith-based growth experiment, described in the subsection below) to an optical density at 750 nm (OD_750_) of 0.2. The volume after inoculation was 70 ml per vessel. This volume ensured that Atmos could be run in its normal mode and that enough biomass would be generated for downstream analyses.

Three of those six vessels were left open to ambient air (4 of the G1/4 holes were filled with cotton plugs; the fifth one was used for the temperature probe). The laboratory is located at sea level and the ambient pressure is ca. 101 kPa. Air in the other three vessels was evacuated down to 100 hPa and replaced with MDA-1, using a tank (provided by Air Liquide) containing 4.000 ± 0.080 vol% of CO_2_, the rest being N_2_ (see Figure 2). The gas in the headspace was renewed (by flushing for 5 min at a rate of ca. 0.1 standard-l min^−1^, at constant pressure) 2 h after starting the experiment, and then every 6 h throughout the experiment. Due to water evaporation, pressure increased slightly following gas renewal, but the pressure regulation system lowered it down to 100 hPa if it deviated by 10% of the target value. The measured total pressure was on average 101.5 ± 0.31 hPa. Light intensity was set to 5 μmol photons m^−2^ s^−1^ per side, temperature to 25 °C, and stirring to 100 rpm. The measured temperature was on average 26.6 ± 0.21 °C over the course of the experiment (set and actual temperatures differed due to unusually high room temperatures).

Triplicate samples from the culture used as a source of inoculum were collected and dried to assess its biomass concentrations. The experiment lasted 10 days, after which cultures under MDA-1 were brought back to ambient atmospheric conditions and all six vessels were disconnected from Atmos. Growth was assessed, and biomass further processed, as described below. Growth conditions are summarized in Table 1 (Experiment I).

**Table 1:** Summary of the conditions in which *Anabaena* sp. was grown for the work presented here.

**Parameter**	**Experiment I (standard medium)**	**Experiment II (regolith)**
Atmospheric conditions	MDA-1; ambient	MDA-1; ambient
Medium	BG11_0_	Regolith in water; BG11_0_; regolith in BG11_0_; water
Volume per sample	70 ml	4 ml
Stirring	100 rpm	None
Average pressure under MDA-1 (measured)	101.5 hPa	103.4 hPa
Temperature (set)	25°C	Ambient
Average temperature (measured)	26.6°C	23.6°C
Duration	10 days	14 days; 21 days; 28 days

*For a comparison between MDA-1 and ambient atmosphere, see Figure 2*.

### 2.4. Regolith-Based Growth Under MDA-1

In order to assess whether MDA-1 would prevent the regolith-dependent growth of *Anabaena* sp., the latter was grown in double-distilled and deionized water containing a simulant of Martian regolith, under either ambient air or MDA-1.

**Figure 4:**
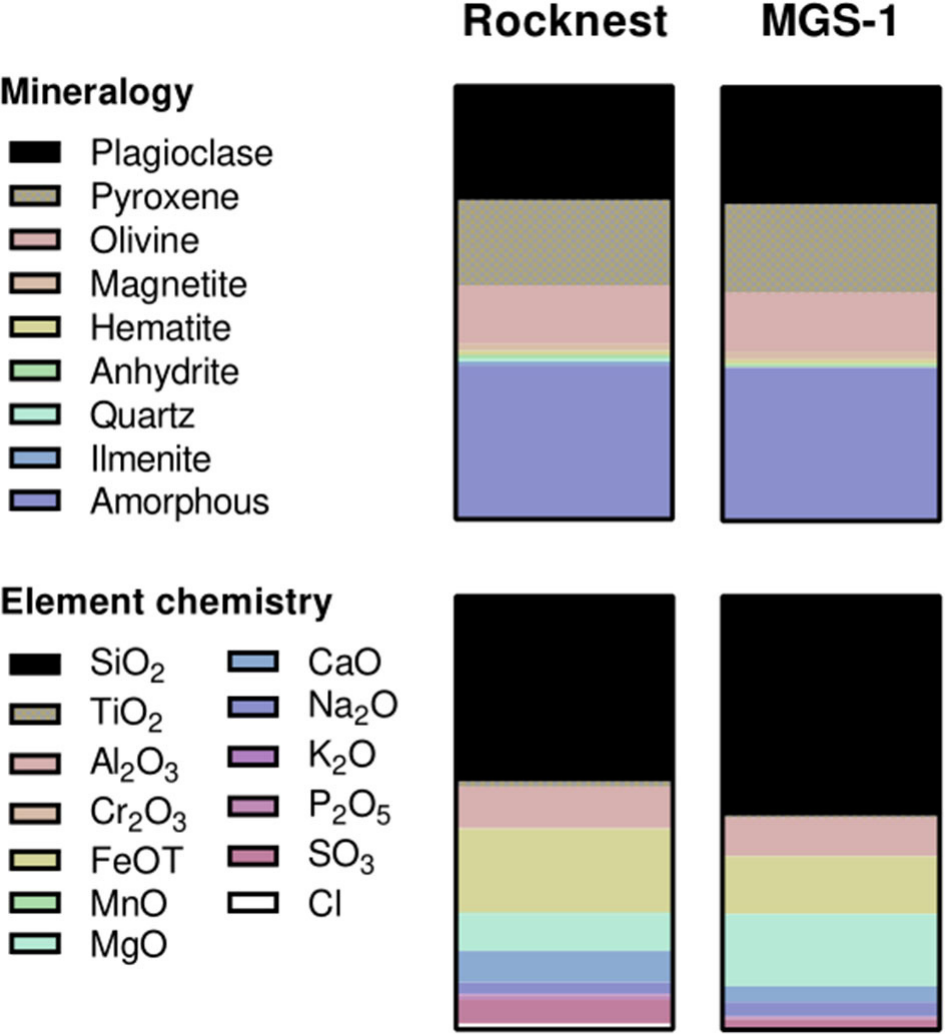
Mineralogy **(top)** and major element chemistry **(bottom)** of Rocknest soil (Achilles et al., 2017) and its simulant, MGS-1 (Cannon et al., 2019). Proportions correspond to weight fractions. Further details on MGS-1 can be found in Cannon et al. (2019).

As a simulant, we used the Mars Global Simulant (MGS-1; Cannon et al., 2019), an analog based on the Rocknest windblown soil at Gale crater (Figure 4). It was obtained from the Center for Lunar and Asteroid Surface Science (Orlando, Florida, USA). Prior to experiments, it was baked at 450 °C for 12 h to degrade organic contaminants.

For this experiment, Atmos was not used in its normal mode: samples were placed in small Petri dishes (diameter: 35 mm) and stacked into vessels. While culture volumes were consequently reduced, and the heating and stirring systems could not be used, this setup enabled the simultaneous exposure of a higher number of samples. Dishes were filled with (i) 4 ml of BG11_0_, (ii) 4 ml of double-distilled and deionized water and 0.8 g of regolith simulant (the regolith-to-water ratio was selected based on previous work by Olsson-Francis and Cockell, 2010), (iii) 4 ml of double-distilled and deionized water, or (iv) 4 ml of BG11_0_ and 0.8 g of regolith simulant. A cyanobacterium culture in late exponential phase was washed twice in water, centrifuged, and resuspended in water to an OD_750_ of ca. 1. A volume of 80 μl was added to each plate. Abiotic controls were prepared that contained either water and regolith simulant, or BG11_0_, but were not inoculated; those were exposed to ambient air only.

Dishes were sealed with Micropore tape (over a quarter of the circumference) and Parafilm (remaining 3 quarters), to limit evaporation without preventing gas circulation, and distributed among six vessels (in triplicate for each vessel). A water reservoir (ca. 40 ml) was added to each vessel to further limit evaporation. The air in half of the vessels was replaced with MDA-1 and renewed daily (see above); the pressure measured inside those vessels was on average 103.4 ± 1.5 hPa over the course of the experiment. The other vessels were left open to ambient air. Light intensity was set to 5 μmol photons m^−2^ s^−1^ per side. Temperature was not controlled (the stack of dishes prevented the use of the heating rubbers and temperature probes) and was, on average, 23.6 ± 0.4 °C over the course of the experiment (measured inside Atmos, outside of but next to a vessel). Growth conditions are summarized in Table 1 (Experiment II).

Two vessels (one per atmospheric condition) were taken out of the experiment after 14, 21, and 28 days, and the amounts of chlorophyll a per dish were determined as described below. The significance of observed differences was assessed using a two-way repeated measures ANOVA followed by Tukey's test, with an adjusted *p*-value threshold at 0.05.

### 2.5. Assessments of Growth

The growth of cyanobacterium samples cultivated for 10 days in standard medium, under ambient atmosphere or MDA-1 (Experiment I in Table 1), was assessed based on OD_750_ and dry weight of biomass. For the latter, we performed 3 measurements per biological replicate and for the inoculum, as follows. Forty-five ml of culture per vessel were split into 3 15-ml Falcon tubes, and washed twice in distilled water and once in double-distilled and deionized water. Pellets were then transferred to pre-weighed sheets of aluminum foil and dried at 60 °C in a drying oven. The final weight of each sample was determined using an analytical balance. Values for OD_750_ and dry weight of biomass at culture onset were calculated from measured values of the inoculum. The significance of the difference between compared pairs of means was assessed using two-tailed *t*-tests, with a *p*-value threshold at 0.05.

As the presence of regolith would have interfered with measurements of dry weight and optical density, growth in samples of the experiment involving MGS-1 (Experiment II in Table 1) was assessed using total amounts of chlorophyll a. Chlorophyll a was extracted with ethanol from the whole of each sample and quantified based on optical density at 665 nm (Ritchie, 2008). In order to assess biomass concentrations from chlorophyll a amounts, the chlorophyll a-to-dry biomass ratio was determined after measuring both values, each in triplicate, for the culture used as an inoculum in Experiment II.

### 2.6. Changes in Cellular Physiology: Heterocyst Spacing, and Carbohydrate and Protein Contents

Samples from *Anabaena* sp. grown for 10 days in standard medium, under either MDA-1 or ambient air (Experiment I in Table 1), were set aside to assess selected parameters associated with cell physiology: the average distance between heterocysts, and the fraction of the biomass represented by carbohydrates and soluble proteins.

For the determination of heterocyst spacing, samples were fixed with 4% paraformaldehyde in phosphate-buffered saline and stored at 4 °C until the following day. The average number of vegetative cells separating heterocysts was then determined under an inverted microscope (Bresser Science IVM 401). We performed 20 measurements per biological replicate. Dividing cells were counted as two when an obvious septum had formed. Images were acquired using a mounted camera (Bresser MikroCam 5.0) and the associated software (MicroCamLabII).

Carbohydrate concentrations were determined by the phenol-sulfuric acid method (DuBois et al., 1956), using D-glucose as the standard. Soluble proteins were extracted by 3 cycles of bead beating (10 min) and cooling down on ice (5 min), after which samples were centrifuged and proteins quantified in the supernatant with the Invitrogen Qubit 4 Fluorometer and the Qubit Protein Assay Kit. For each biological replicate, we quantified proteins from 3 subsamples and carbohydrates from 2 (due to the loss of part of the samples).

The significance of the difference between compared pairs of means was assessed using two-tailed t-tests, with a *p*-value threshold at 0.05.

### 2.7. Growth of *E. coli* in Cyanobacterium-Based Medium

The remaining biomass from *Anabaena* sp. grown for 10 days in standard medium, under either ambient atmosphere or MDA-1 (Experiment I in Table 1), was pooled by atmospheric condition and used to prepare cyanobacterium-based media as described by Verseux (2018). Dry biomass was ground with a pestle in a liquid nitrogen-cooled mortar, weighed, and resuspended in double-distilled and deionized water to reach a biomass concentration of 25 g l^−1^. Samples were incubated for 2 h at room temperature under mild agitation, then centrifuged (7,000 rcf, 10 min). Supernatants were pre-filtered with 1 μm glass-fiber filters (Acrodisc, Pall Corporation) and filtered with 0.22 μm cellulose ester filters (Millex-GS, Merck Millipore). The filtrate was stored at −20°C until the following day.

As a model secondary consumer, we used *E. coli* W, a well-characterized strain able to use sucrose as a carbon source and previously shown to reach particularly high cell concentrations (about twice as high as obtained with *E. coli* K-12 MG1655) in a lysate of *Anabaena* sp. PCC7120 (Verseux, 2018). LB medium was inoculated with a single colony and incubated overnight at 37 °C under agitation. This pre-culture was used to inoculate a fresh culture, which was incubated under the same conditions until it reached the stationary phase. It was then washed 3 times in saline, centrifuged, and resuspended in 3 ml of saline. This suspension was used to inoculate saline (2 sets), both cyanobacterium-based media, and LB medium. Cell density in the inoculum, determined based on colony counts (see below), was 1.0 × 10^9^ ± 1.1 × 10^8^ cfu ml^−1^; this value was used to calculate cell concentrations at culture onset. Each set of conditions was prepared in triplicate. Samples from one of the saline sets were immediately diluted serially and spread on agar plates. The other samples were incubated overnight at 37 °C, under agitation, then serially diluted and spread on agar plates. All plates were incubated overnight at 37 °C, after which colonies were counted to assess *E. coli*'s initial and final cell concentrations. The significance of the difference between compared pairs of means was assessed using an ordinary one-way ANOVA followed by Tukey's test, with an adjusted *p*-value threshold at 0.05.

### 2.8. Statistical Analysis

Statistical tests were performed using GraphPad Prism version 8.4.3 for Windows, by GraphPad Software (San Diego, California).

## 3. Results

### 3.1. *Anabaena* sp. Grew Vigorously Under 100 hPa of a 96% N_2_, 4% CO_2_ Atmosphere

The growth of *Anabaena* sp. cultivated for 10 days under MDA-1 or ambient atmosphere, in BG11_0_, was assessed by measuring OD_750_ and weighing dry biomass.

**Figure 5:**
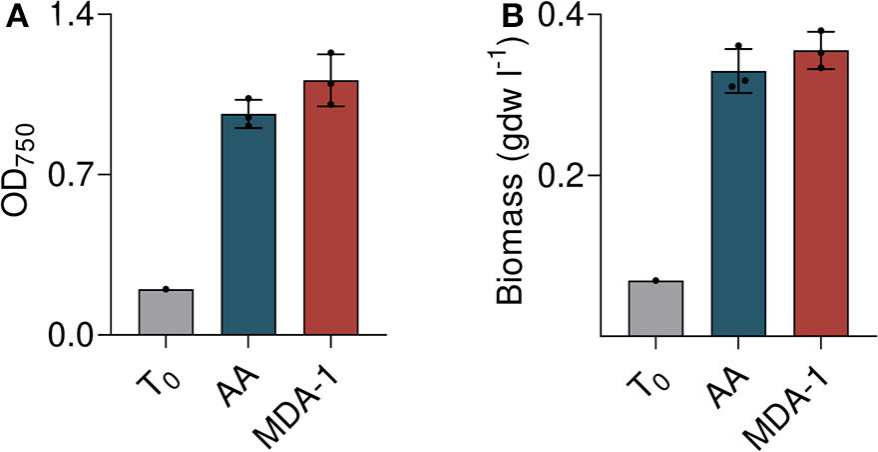
Growth of *Anabaena* sp. under ambient atmosphere (AA) and MDA-1, at cultivation onset (T_0_; calculated from measured values of the inoculum) and after 10 days, as assessed by optical density at 750 nm (OD_750_; **A**) and biomass concentration **(B)** after correction for evaporation. Columns and error bars represent mean values of, and standard deviations across, three biological replicates. Values for biological replicates (dots) are the average of three measurements each. Differences between both atmospheric conditions are non-significant (two-tailed *t*-tests, *p* > 0.05).

Both methods were consistent and showed vigorous growth of cultures under MDA-1 (Figure 5). Starting from an OD_750_ of 0.20 and a biomass concentration of 0.07 g dry weight per liter (gdw l^−1^), it reached an OD_750_ of 1.26 ± 0.13 and a biomass concentration of 0.40 ± 0.026 gdw l^−1^, which is not significantly different from that of cells grown under ambient atmosphere (OD_750_ = 1.03 ±0.08, biomass concentration = 0.35 ± 0.03 gdw l^−1^). Evaporation was slightly higher under MDA-1 than under ambient atmosphere: final volumes were 61.7 ± 0.6 vs. 65.7 ml. Corrected for evaporation (i.e., multiplied by the ratio of final volume-to-initial volume), OD_750_ was 1.11 ± 0.11 and 0.96 ± 0.06 for cultures grown under MDA-1 and ambient atmosphere, respectively, and biomass concentration 0.36 ± 0.03 and 0.33 ± 0.03 gdw l^−1^. The differences are not significant.

### 3.2. Growth Under MDA-1 Induced Physiological Changes, as Illustrated With Reduced Heterocyst Spacing and Reduced Concentrations of Soluble Proteins

Subsamples from *Anabaena* sp. grown for 10 days in standard medium were used to estimate whether MDA-1 (as opposed to ambient air) would affect selected parameters associated with cell physiology, namely heterocyst spacing and the fraction of biomass represented by soluble proteins and carbohydrates.

**Figure 6:**
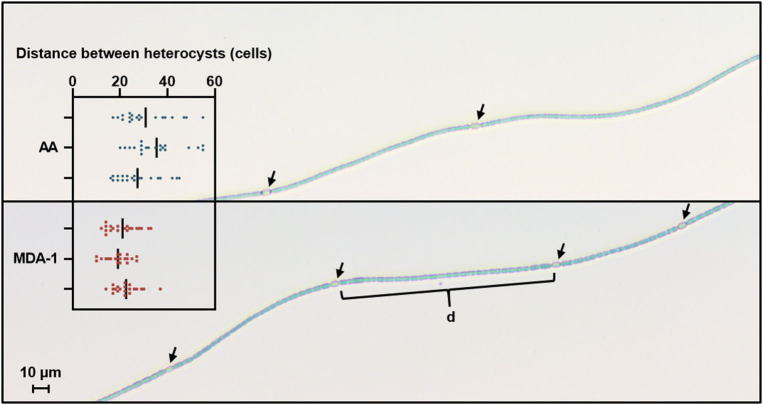
Microscopy images of *Anabaena* sp. filaments grown for 10 days under ambient atmosphere (AA, **top**) or MDA-1 **(bottom)**, showing heterocysts (arrows) and vegetative cells. Bars in the scatter plot give the distance between heterocysts, defined as the average number of cells in the interval (d) separating adjacent heterocysts along a filament, for three biological replicates. Symbols show values for 20 measurements per biological replicate. Overall averages are 31.2 ± 4.0 and 20.9 ± 1.7 cells for AA and MDA-1, respectively. The difference is significant (two-tailed *t*-test, *p* < 0.05).

Heterocyst spacing was assessed by counting, under an optical microscope, the number of vegetative cells separating heterocysts along filaments (Figure 6). Results showed significantly lower distances between heterocysts in filaments grown under MDA-1 (20.9 ± 1.7 cells) than in those grown under ambient atmosphere (31.2 ± 4.0 cells).

Carbohydrates and soluble proteins were extracted and quantified using spectrophotometry-based methods. Results (Table 2) showed a reduced fraction represented by soluble proteins (18.3 ± 1.7 vs. 22.4 ± 1.5%), but no significant difference in carbohydrate contents (22.1 ± 1.5 vs. 21.8 ± 1.5%).

**Table 2:** Carbohydrate and soluble protein contents in biomass of *Anabaena* sp. grown for 10 days under ambient atmosphere (AA) or MDA-1.

**Atmosphere**	**Carbohydrates**	**Soluble proteins**
AA	21.8 (20.1–22.8)	22.4 (21.0–24.0)
MDA-1	22.1 (20.4–23.2)	18.3 (17.2–20.3)

*Data are expressed as percentage of dry mass (average and range) over three biological replicates. Values for biological replicates are the average of 2 (carbohydrates) or 3 (proteins) measurements each. The difference in protein contents, but not in carbohydrate contents, is significant (two-tailed t-test, p < 0.05)*.

### 3.3. *Anabaena* sp. Could Grow Under MDA-1 When a Regolith Simulant Was Used as a Source of Metal Nutrients

In order to assess whether MDA-1 (as opposed to ambient atmosphere) would prevent the regolith-dependent growth of *Anabaena* sp., we cultivated cells for 14, 21, and 28 days in dishes containing either standard medium (BG11_0_) or water and MGS-1, an analog of Martian regolith. Changes in amounts of chlorophyll a were used as a proxy for growth.

Positive controls were grown in standard medium (BG11_0_), with and without MGS-1. Abiotic controls (containing either water and MGS-1, or BG11_0_, but no cells) were used to confirm that growth media did not interfere with absorbance measurements (following extraction, absorbance was equal to that of the ethanol blank, for all time points). Additional negative controls consisted in cells incubated in water only.

**Figure 7:**
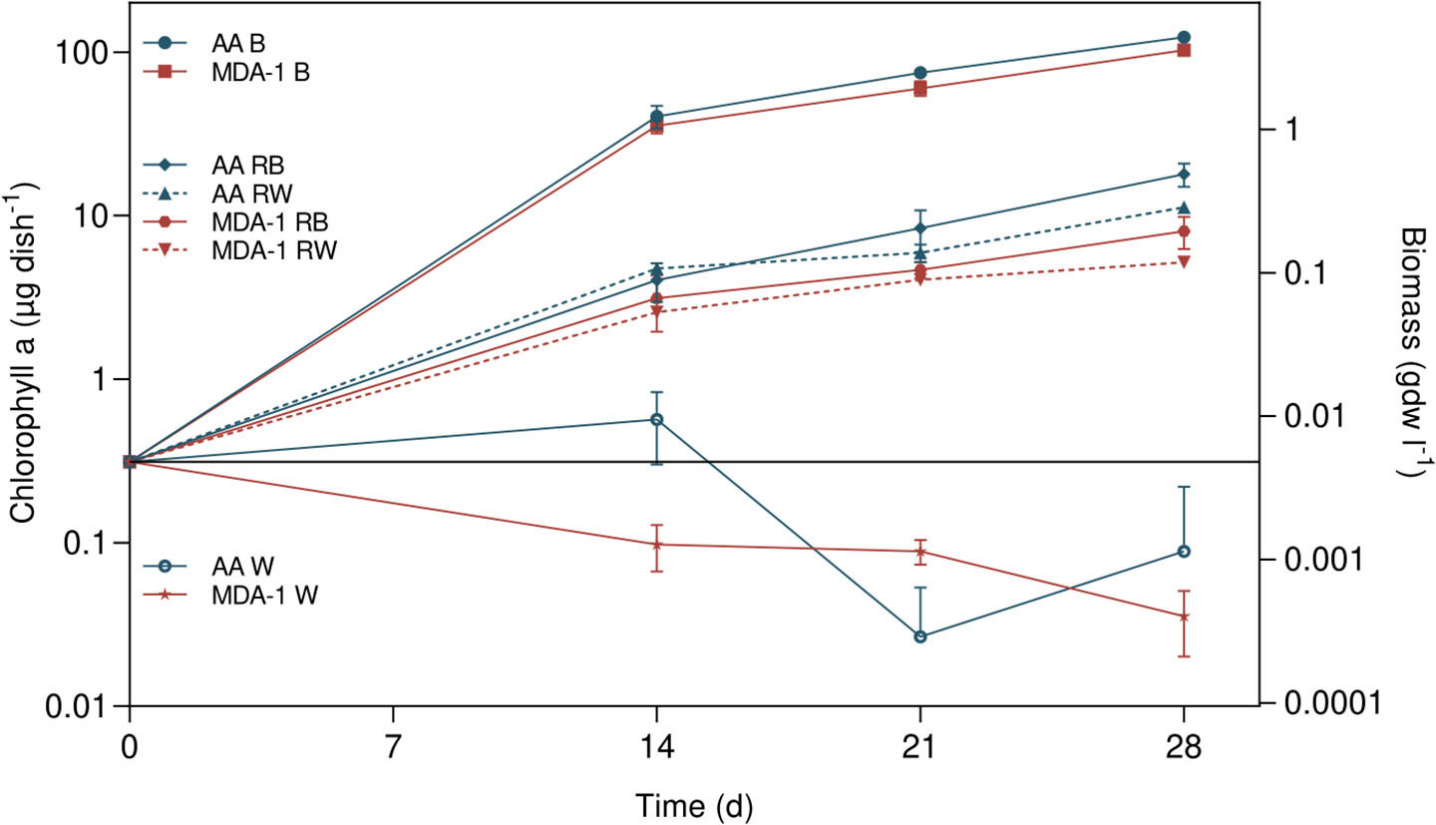
Amounts of chlorophyll a in *Anabaena* sp. samples, at culture onset (calculated from cell concentrations in the inoculum) and after 14, 21, and 28 days, under either ambient atmosphere (AA) or MDA-1. Samples were cultivated in either BG11_0_ (B), BG11_0_ containing 0.2 g ml^−1^ of MGS-1 regolith simulant (RB), double-distilled and deionized water containing 0.2 g ml^−1^ of MGS-1 (RW), or water (W). Biomass concentrations were calculated from chlorophyll a amounts, using the determined chlorophyll a-to-biomass ratio, for a volume of 4 ml. Symbols represent mean values of three biological replicates. Error bars show standard deviations of chlorophyll a amounts across biological replicates. Differences between atmospheric conditions are not significant in BG11_0_ (Tukey test, adjusted *p* > 0.05), but they are in water supplemented with MGS-1 after 14 and 28 days (*p* < 0.01). No chlorophyll a was detected in abiotic controls (BG11_0_, or water containing 0.2 g ml^−1^ of regolith, non-inoculated).

Growth took place in all inoculated samples containing BG11_0_, MGS-1 in BG11_0_, or MGS-1 in water: amounts of chlorophyll a, initially of 0.3 μg of chlorophyll a per dish (μg chl. a dish^−1^), increased over time (Figure 7). On the contrary, final chlorophyll a amounts in inoculated water without MGS-1 were lower than amounts at the onset of the experiment.

At all three time points, chlorophyll a amounts were significantly higher (by an order of magnitude) in inoculated samples containing BG11_0_ than in those containing MGS-1 in water: in BG11_0_, they reached 123.6 ± 7.2 μg chl. a dish^−1^ under ambient air and 102.9 ± 5.5 μg chl. a dish^−1^ under MDA-1, while with MGS-1 in water they reached 11.2 ± 0.6 μg chl. a/dish^−1^ under ambient air and 5.2 ± 0.2 μg chl. a dish^−1^ under MDA-1. The differences in amounts of chlorophyll a between samples incubated under MDA-1 and those incubated under ambient air were not significant when the medium was BG11_0_. However, they were significant after 14 and 28 days when cells were grown in water with MGS-1.

Growth in samples containing MGS-1 in BG11_0_ was significantly lower than in samples containing BG11_0_ but no regolith: amounts of chlorophyll a in the former reached 17.9 ± 2.9 μg chl. a/dish^−1^ under ambient air and 8.0 ± 1.8 μg chl. a/dish^−1^ under MDA-1, which is not significantly different from amounts in samples grown with MGS-1 in water under the corresponding atmospheres.

### 3.4. Cyanobacterial Growth in MDA-1 Did Not Reduce the Suitability of *Anabaena* sp. Extracts as a Substrate for *E. coli*

After determining that MDA-1 could support vigorous cyanobacterial growth in standard medium, and did not prevent *Anabaena* sp.'s growth when MGS-1 was used as a nutrient source, we proceeded to determine whether the resulting biomass would be less suitable as a substrate for downstream modules of BLSS. We hypothesized that such a drawback could result from changes in biomass composition, illustrated by the reduced fraction of soluble proteins.

Dried biomass from *Anabaena* sp. grown under either ambient atmosphere or MDA-1 was thus ground, suspended in water at a concentration of 25 g l^−1^, filtered, and used as a substrate for growing of *E. coli* W. Control cultures (positive and negative, respectively) were prepared in LB medium and saline solution.

**Figure 8:**
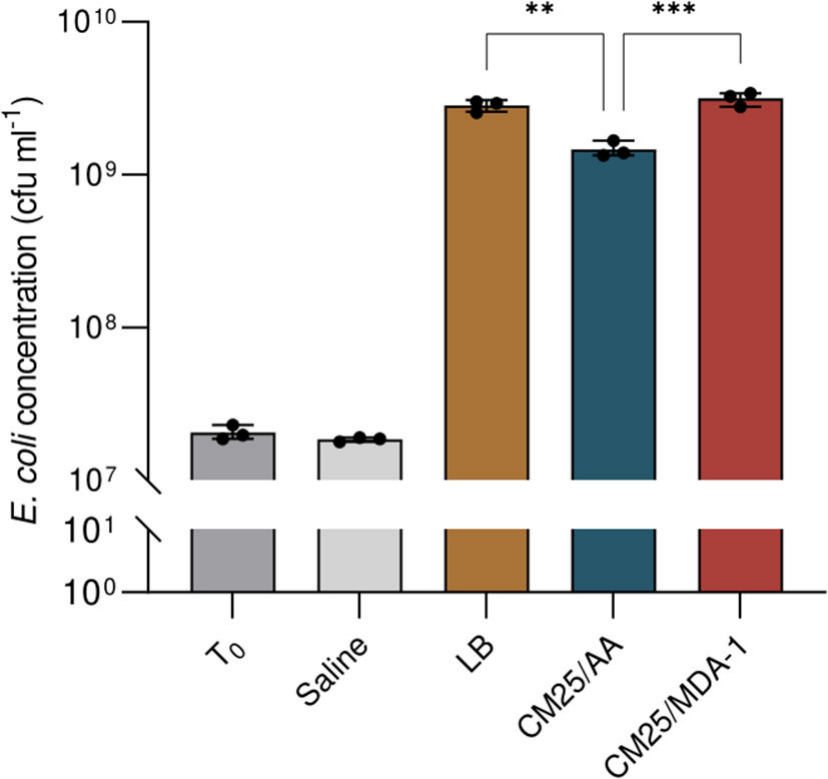
Cell concentrations of *E. coli* at culture onset (T_0_) and after overnight incubation in saline solution (Saline), standard medium (LB), or a filtered lysate of *Anabaena* sp. previously grown under either ambient atmosphere (CM25/AA) or MDA-1 (CM25/MDA-1). Columns and error bars represent mean values of, and standard deviations across, three biological replicates. Dots show values for individual biological replicates. The cell concentration in CM25/AA differs significantly from those in CM25/MDA-1 and LB (Tukey test; ***, adjusted *p* < 0.001; **, adjusted *p* < 0.01); differences between CM25/MDA-1 and LB are non-significant (*p* > 0.05). cfu, colony forming units.

Results are shown in Figure 8. After overnight incubation, cell concentrations in the filtered lysates (originally at 2.0 × 10^7^ ± 2.3 × 10^6^ cfu ml^−1^) were in the same order of magnitude as in LB medium (where they reached 2.8 × 10^9^ ± 1.4 × 10^8^ cfu ml^−1^). Final cell densities were significantly higher when the lysate was prepared from cyanobacteria grown under MDA-1 (3.2 × 10^9^ ± 3.2 × 10^8^ cfu ml^−1^) rather than under ambient atmosphere (1.5 × 10^9^ ± 1.8 × 10^8^ cfu ml^−1^).

## 4. Discussion

Our results suggest that a photobioreactor deployed on the Martian surface, as part of a CyBLiSS, could rely on an N_2_/CO_2_ atmosphere at reduced pressure. First, an atmosphere of 96% N_2_ and 4% CO_2_ at a total pressure of 100 hPa (MDA-1) supported the vigorous growth of *Anabaena* sp. Second, the resulting biomass seems suitable as a substrate for downstream BLSS modules, as shown here with the heterotrophic bacterium *E. coli*. Third, MDA-1 did not prevent the utilization by *Anabaena* sp. of an analog of Martian regolith, substantiating the hypothesis that cyanobacteria could be grown on Mars using nitrogen and carbon from the atmosphere and obtaining mineral nutrients from the regolith. While our results should not be mistaken for a quantitative estimate of yields that would be obtained on site from a mature BLSS, they demonstrate that a mixture of gases that eases engineering constraints (low pressure and gases available on site) can meet the requirements dictated by biology.

When selecting a model cyanobacterium, two closely related genera were considered: *Nostoc* and *Anabaena*. Species from both can fix nitrogen and some grow at high rates (by cyanobacterial standards), can use rock substrates efficiently (Arai et al., 2008; Olsson-Francis and Cockell, 2010; Olsson-Francis et al., 2012), form akinetes which are highly resistant to conditions found in space and on Mars (Olsson-Francis et al., 2009), produce H_2_ (e.g., Abed et al., 2009), can be genetically engineered, and/or have been shown to be edible—all of which are desirable properties for CyBLiSS (Verseux et al., 2016a). Within those genera, we chose *Anabaena* sp. PCC 7938 for its growth rates, robustness, culture homogeneity, and after preliminary results showing efficient growth when relying on various Moon and Mars regolith simulants (not shown).

While various setups have been assembled for the study of microorganisms at pressures lower than ambient (reviewed in Schwendner and Schuerger, 2020; Verseux, 2020a), no photobioreactor was available that could provide accurate and stable atmospheric conditions at 100 hPa or below, especially to multiple, individually-controlled vessels. We consequently developed Atmos, a low-pressure photobioreactor. It includes nine one-liter culture vessels that can be programmed for commonly-adjustable growth parameters (light intensity, stirring speed, and temperature) as well as for atmospheric pressure and composition. Though designed primarily for investigations on cyanobacterial behavior under atmospheres relevant to Mars-specific BLSS, Atmos can be used for other studies related to the physiology of microorganisms (as well as small plants) at low pressure. This area bears relevance to fields such as in-habitat BLSS, planetary protection, habitability, ecopoiesis, and aerobiology (Paul and Ferl, 2007; Schwendner and Schuerger, 2020; Verseux, 2020a). We intend for our device to support the astrobiology and BLSS communities through collaborative projects.

Atmos was used, first, for testing the hypothesis that an atmosphere derived from gases present in the Martian atmosphere (N_2_ and CO_2_), at low pressure, could efficiently support the growth of diazotrophic cyanobacteria relying on it as a source of carbon and nitrogen. We grew *Anabaena* sp. under ambient air and MDA-1, in 70 ml of nitrate-free BG11 medium (BG11_0_) agitated with a stir bar. Our reasoning behind the design of MDA-1 was as follows. A pCO_2_ of 4 hPa was expected not to be limiting, even with an abundant nitrogen source (Murukesan et al., 2015); this value would be an advantage over Earth-ambient air where CO_2_ is scarce (see Figure 2 for a comparison between Earth's atmosphere and MDA-1). The limiting gas would thus be N_2_: at a non-limiting partial pressure (above ca. 500 hPa; see MacRae, 1977; Klingler et al., 1989; Silverman et al., 2019), it would conflict with the requirement of maintaining a low total pressure. Our compromise was a pN_2_ of 96 hPa, which brings the total pressure to 100 hPa—roughly the value from which pressure itself is thought not to largely affect bacteria (Schuerger et al., 2013; Verseux, 2020a). The combined effect of the high (compared to Earth-ambient) pCO_2_, low pN_2_, and low total pressure was such that MDA-1 supported vigorous cyanobacterial growth: after 10 days of cultivation, biomass concentrations were similar to those obtained under ambient air.

Even though we chose a cultivation time of 10 days so that cultures would not be in the stationary phase (to reflect growth rates rather than final concentrations), a single sampling event cannot be used to conclude that growth dynamics were unaffected by the change in atmospheric conditions. Cultures brought from ambient air to MDA-1 have to acclimate to the latter. This acclimation seems to include an increase in heterocyst frequency: in *Anabaena* sp., nitrogen fixation is separated from photosynthesis as nitrogenases (the enzymes responsible for nitrogen fixation) are inactivated by oxygen; it occurs in specialized cells called heterocysts. As those do not fix carbon, an efficient distribution of nutrients along a filament requires a tight control of heterocyst patterns. It was for instance shown that heterocyst spacing (i.e., the number of vegetative cells that separate two adjacent heterocysts on a filament; see Figure 6) in *Anabaena cylindrica* decreases with pN_2_ in the absence of fixed nitrogen (Silverman et al., 2019). Similarly, we observed a decreased heterocyst spacing in *Anabaena* sp. under MDA-1. Also consistent with an initial need for acclimation, visual inspections of the vessels during growth suggested lower densities under MDA-1 in the first few days, before they increased at a rate high enough to meet the density of the samples under ambient atmosphere. Those observations are, however, anecdotal. Documenting growth dynamics under accurately controlled conditions (i.e., using the normal mode of Atmos, as opposed to the stacks of Petri dishes used for regolith-related experiments) is something we aim for in the future; presently, we simply conclude that a gas phase derived from the Martian atmosphere, at a pressure much below Earth-ambient at sea level, can adequately support the diazotrophic, autotrophic growth of cyanobacteria.

This finding could contribute significantly to the feasibility of CyBLiSS. The low pressure would reduce engineering constraints related to the inside/outside pressure difference of a photobioreactor deployed on the Martian surface, while the N_2_/CO_2_ composition would allow for the atmosphere to be produced from locally available gases, with minimal processing, using systems based on technologies routinely used by industry on Earth (Ley et al., 2000; Muscatello et al., 2011). A high level of gas purity is not needed, and at least the separation of CO_2_ from other gas components is likely to be performed as well for other *in situ* resource utilization processes (Starr and Muscatello, 2020). CO_2_ and N_2_ could then be mixed at the desired ratio, and the total pressure brought to the target value.

In a CyBLiSS as previously described (Verseux et al., 2016a), nutrients not provided to cyanobacteria from the atmosphere would come from weathering Mars's regolith in water mined on site. This regolith is mostly basaltic; its composition is known, mostly, from spectroscopic data from orbiting spacecraft, the study of Martian meteorites, and *in situ* analyses at hundreds of locations around landers' landing sites and along rovers' paths (e.g., Clark et al., 1982; Gellert et al., 2006; Ming et al., 2008; McLennan et al., 2014; Vaniman et al., 2014; Siebach et al., 2017; Rampe et al., 2020). Nitrogen (presumed to belong to nitrates) was detected in aeolian samples and mudstone deposits, but at concentrations which are too low for supporting strong microbial metabolism: nitrates would represent from below 0.01 wt% to ca. 0.1 wt% of the tested samples (Stern et al., 2015). Organic carbon was detected as well (Eigenbrode et al., 2018; Franz et al., 2020; Szopa et al., 2020) but in debated and presumably low amounts, possibly because organics are largely degraded at the surface by radiation and oxidizers (whether higher, exploitable amounts could be found in the subsurface is unknown). However, both carbon and nitrogen can be provided as CO_2_ and N_2_, and all required elements which are not found in the atmosphere or water (P, S, K, Mg, Na, Ca, Fe, Mn, Cr, Ni, Mo, Cu, Zn, etc.) have been detected in Mars's regolith (for a discussion in the context of biology, see Cockell, 2014). While no sample from Mars has so far been returned, it was demonstrated that *Anabaena* and *Nostoc* spp. can grow using volcanic rocks analogous to Martian regolith (Arai et al., 2008; Olsson-Francis and Cockell, 2010; Olsson-Francis et al., 2012; Verseux, 2018). Those tests, however, were performed under ambient atmosphere, and combinations of stressors often have synergistic effects (e.g., Harrison et al., 2013). As a particularly fitting example: while *Serratia liquefaciens* could grow both (i) in a nutritive medium mixed with any of 3 Mars analog soils, at 30 °C, under ambient air and (ii) at circa 0 °C, under 7 hPa of a CO_2_-enriched anoxic atmosphere (low-PTA conditions), it failed to grow in the presence of the soils under low-PTA conditions (Schuerger et al., 2020).

We consequently assessed whether MDA-1 would affect the regolith-dependent growth of *Anabaena* sp. This was done by growing cyanobacteria in small Petri dishes (without agitation) containing 4 ml of water and 0.8 g of MGS-1, an analog of Martian regolith based on the Rocknest windblown soil at Gale crater (Cannon et al., 2019). This analog was chosen because of its high fidelity in terms of mineral and chemical properties (Figure 4), and its representing a widespread regolith unit, which make it more relevant to biology experiments than other widely available simulants such as, for instance, JSC Mars-1 and MMS derivatives (Cannon et al., 2019; Eichler et al., 2020). Contrary to the latter two (Wamelink et al., 2014; Guinan, 2018), this simulant was shown to be unsupportive of plant growth, even with nutrient supplementation (Eichler et al., 2020). *Anabaena* sp., on the other hand, could grow in water containing MGS-1, without any nutrient supplementation, under either test atmosphere.

Regolith-based growth was slower than growth in BG11_0_. This result, consistent with previous studies (Arai et al., 2008; Olsson-Francis and Cockell, 2010; Olsson-Francis et al., 2012; Verseux, 2018), can be explained in part by the following: while all nutrients not found in the atmosphere are available at onset in BG11_0_, their release from MGS-1 depends upon rock dissolution. Release rates from basaltic rocks cannot be predicted based on environmental conditions and bulk elemental composition alone: they depend on a complex set of interrelated factors such as primary mineral composition, microbe-basalt interactions, and the formation of secondary minerals (e.g., Wu et al., 2007; Olsson-Francis et al., 2012; Byloos et al., 2018). Other mechanisms may be at play, as suggested by the decrease in growth rates caused by MGS-1 in BG11_0_ (though synergistic effects resulting from the combination of BG11_0_ and MGS-1, such as elements reaching above-optimal concentrations, cannot be ruled out); it is likely that among them is a reduction in light availability due to regolith grains. Although low growth rates in regolith would be a limitation for CyBLiSS, productivity may be enhanced by the optimization of culture conditions and through bio-engineering (Verseux et al., 2016b).

Using MDA-1 rather than ambient air did not significantly affect growth in the BG11_0_ controls. It did, however, reduce it significantly when cells relied on regolith as a substrate, leading to approximately half the amounts of chlorophyll a after 28 days. We currently cannot conclude whether this is due to a period of acclimation to MDA-1, not compensated for after 28 days due to the slower growth rates in regolith, and/or to a synergistic effect of MDA-1 and a dependence on regolith for nutrients. In a preliminary experiment (not shown) where temperatures were higher (28.6 ± 1.9 °C vs. 23.6 ± 0.4 °C here), resulting in their amounts of chlorophyll a after 28 days being close to 3 times larger, cultures in water and MGS-1 under MDA-1 did not differ significantly from those under ambient atmosphere. It may however be that both cultures had reached a stationary phase (no intermediate time points were studied), thus reflecting final concentrations rather than growth rates. Further investigations are needed on the synergistic effects of both factors (atmospheric conditions and dependence on regolith) and on the resulting dynamics, including a potential acclimation and adaptation.

Besides cyanobacteria's abilities to grow using compounds available in Mars's regolith and atmosphere, a key assumption behind CyBLiSS is that the resulting cultures could be used for feeding other biological systems that cannot rely as directly as cyanobacteria on Mars's natural resources (because they cannot access mineral nutrients from the regolith, require organics, and/or cannot metabolize gases from the Martian atmosphere). It was previously shown that soluble extracts from the cyanobacterium *Anabaena* sp. PCC 7120, grown under ambient air, could support the proliferation of at least some species of heterotrophic bacteria including *E. coli* MG1655, *E. coli* W, *Bacillus subtilis* 168, and *B. subtilis* SCK6 (Verseux, 2018).

We wondered whether this transfer of nutrients could be affected by potential changes in cyanobacterium biomass caused by a modified atmosphere: differences in culture conditions (including carbon and nitrogen availability) can lead to different biomass compositions in microalgae (see Jiang et al., 2012; Juneja et al., 2013; Mou et al., 2017). As an example, growing *A. cylindrica* under a low-pressure, high-CO_2_ atmosphere (fixed nitrogen was provided in the medium) caused an increase in the carbohydrate contents, and a decrease in the protein contents, of its biomass (K. Lehto, reported as unpublished results in Verseux et al., 2016a). Similarly, decreased protein contents and increased carbohydrate contents were reported for *Arthrospira platensis* grown under high pCO_2_ at ambient total pressure (Gordillo et al., 1998) and in various microalgae under nitrogen limitation (e.g., Lynn et al., 2000). A slight decrease in soluble protein contents occurred under MDA-1 as well, though the increase in carbohydrates was non-significant. A detailed analysis of how MDA-1 changes cellular composition was beyond the scope of the present study: we rather focused on whether those changes could interfere with the use of *Anabaena* sp. as a source of nutrients for downstream modules of BLSS. We dried, lysed, and resuspended in water (at a concentration of 25g l^−1^) cyanobacterial biomass from cultures grown under MDA-1 and ambient atmosphere. The solutions were filtered, and the filtrates used to grow *E. coli*. After overnight incubation, cell concentrations in the filtrates were in the same order of magnitude as in LB medium, and were higher when the cyanobacterial biomass was grown under MDA-1 than when it was grown under ambient atmosphere. Thus, any change in cyanobacterium biomass composition appeared not to be detrimental to secondary consumers. They were even beneficial, presumably because changes in cyanobacterial metabolism led to an increase in the fraction of soluble compounds which *E. coli* can utilize. A thorough characterization of cyanobacterium-based media prepared from cells grown under both atmospheric conditions would help test this assumption.

Taken as a whole, our results suggest that a CyBLiSS photobioreactor on the Martian surface could rely on a mixture of gases extracted from the local atmosphere and brought to a tenth of Earth's sea-level pressure. However, the values we presented should not be mistaken for quantitative estimates of a CyBLiSS's productivity. First, Atmos was designed to compare behavior under different atmospheric conditions, not to maximize efficiency: other parameters, such as lighting or gas transfer, were not optimized. Conditions that optimize growth on regolith may, in addition, differ from those in a standard medium. Second, MDA-1 may not be the atmosphere selected, ultimately, for cyanobacterium cultivation modules. Optima will depend on yet-unknown parameters such as mission architecture, photobioreactor design, and weight assigned to decision criteria (e.g., lower payload mass vs. higher biomass productivity). Besides, further data may help fine-tune the combination of total pressure, pCO_2_, and pN_2_. Third, the strain we used for this study is likely not that which would be used on Mars: a number of candidates from various genera should be identified and compared, and possibly tailored for the task using bio-engineering. Fourth, growth dynamics were not fully documented; doing so may lead to better estimates. As an example, if an extended lag phase is caused by cyanobacterial acclimation to MDA-1, a continuous cultivation (or drawing an inoculum from a culture previously grown under MDA-1) would lead to higher average growth rates than could be deduced from the present data. It should however be noted that, when cyanobacteria are grown on regolith, those advantages may be offset by the limiting factor being metal nutrients (or light availability) rather than atmospheric carbon or nitrogen. Fifth, major differences exist between MGS-1 and the soil it simulates. One example is the absence of perchlorates in the former, which have been detected and quantified at several locations on Mars (Hecht et al., 2009; Kounaves et al., 2014; Sutter et al., 2016). Perchlorates are likely ubiquitous at the surface, and how their concentration changes with depth is unknown (Carrier, 2017). Concentrations of perchlorates that would result from using 0.2 g ml^−1^ of regolith with perchlorate contents roughly similar to those found at the Phoenix landing site (Kounaves et al., 2014; Fang et al., 2015) affected, but did not prevent, the growth of *Anabaena* sp. PCC 7120 (Verseux, 2018). However, other highly oxidizing compounds are likely present in the regolith (Lasne et al., 2016). Besides, though soils similar to the Rocknest deposit (on which MGS-1 is based) seem to be found throughout Mars, more dissimilar regolith can be found as well. Some regolith units (and their combinations) may be identified that better support cyanobacterial growth, either as a main substrate or as a supplement to other regolith units.

Future research will aim at refining the design of CyBLiSS, with investigations pertaining to (i) the effects of total pressure and low pN_2_ on cyanobacteria, (ii) the use of regolith as a substrate, and (iii) the transfer of nutrients from cyanobacteria to organisms in downstream BLSS modules. We expect the resulting data to facilitate an assessment of CyBLiSS based on established standards (Levri et al., 2003; Brunet et al., 2010) and a comparison with its alternatives.

## Data Availability Statement

The raw data supporting the conclusions of this article will be made available by the authors, without undue reservation.

## Author Contributions

CV conceived the study. CV, CH, JD, MD, MS, and MA designed and built Atmos. CV and TR performed the experiments. CV, CH, and MA wrote the manuscript. All authors reviewed the manuscript.

## Conflict of Interest

The authors declare that the research was conducted in the absence of any commercial or financial relationships that could be construed as a potential conflict of interest.

## Abbreviations

AA: ambient atmosphere
BG11_0_: nitrate-free BG11 medium
BLSS: bioregenerative life-support system
cfu: colony forming units
CyBLiSS: cyanobacterium-based life-support system
gdw: gram dry weight
MDA-1: Mars-derived atmosphere 1
MGS-1: Mars Global Simulant
OD_750_: optical density at 750 nm
pCO_2_: partial pressure of CO_2_
pN_2_: partial pressure of N_2_.
